# Suitability of a Cellulose-Based Nanomaterial for the Remediation of Heavy Metal Contaminated Freshwaters: A Case-Study Showing the Recovery of Cadmium Induced DNA Integrity Loss, Cell Proliferation Increase, Nuclear Morphology and Chromosomal Alterations on *Dreissena polymorpha*

**DOI:** 10.3390/nano10091837

**Published:** 2020-09-14

**Authors:** Patrizia Guidi, Margherita Bernardeschi, Mara Palumbo, Massimo Genovese, Vittoria Scarcelli, Andrea Fiorati, Laura Riva, Carlo Punta, Ilaria Corsi, Giada Frenzilli

**Affiliations:** 1 Section of Applied Biology and Genetics and INSTM Local Unit, Department of Clinical and Experimental Medicine, University of Pisa, 56126 Pisa, Italy; margherita.bernardeschi@for.unipi.it (M.B.); m.palumbo@studenti.unipi.it (M.P.); vittoria.scarcelli@unipi.it (V.S.); giada@biomed.unipi.it (G.F.); 2 Department of Experimental and Clinical Biomedical Sciences “Mario Serio”, University of Florence, 50121 Florence, Italy; massimo.genovese@student.unisi.it; 3 Department of Chemistry, Materials, and Chemical Engineering “G. Natta” and INSTM Local Unit, Politecnico di Milano, 20131 Milano, Italy; andrea.fiorati@polimi.it (A.F.); laura2.riva@polimi.it (L.R.); carlo.punta@polimi.it (C.P.); 4 Department of Physical, Earth and Environmental Sciences and INSTM Local Unit, University of Siena, 53100 Siena, Italy; ilaria.corsi@unisi.it

**Keywords:** DNA damage, micronucleus, nuclear morphology alteration, cellular proliferation, cadmium, zebra mussel (*Dreissena polymorpha*), polysaccharide-based nanosponge, nanoremediation

## Abstract

The contamination of freshwaters by heavy metals represents a great problem, posing a threat for human and environmental health. Cadmium is classified as carcinogen to humans and its mechanism of carcinogenicity includes genotoxic events. In this study a recently developed eco-friendly cellulose-based nanosponge (CNS) was investigated as a candidate in freshwater nano-remediation process. For this purpose, CdCl_2_ (0.05 mg L^−1^) contaminated artificial freshwater (AFW) was treated with CNS (1.25 g L^−1^ for 2 h), and cellular responses were analyzed before and after CNS treatment in *Dreissena polymorpha* hemocytes. A control group (AFW) and a negative control group (CNS in AFW) were also tested. DNA primary damage was evaluated by Comet assay while chromosomal damage and cell proliferation were assessed by Cytome assay. AFW exposed to CNS did not cause any genotoxic effect in zebra mussel hemocytes. Moreover, DNA damage and cell proliferation induced by Cd(II) turned down to control level after 2 days when CNS were used. A reduction of Cd(II)-induced micronuclei and nuclear abnormalities was also observed. CNS was thus found to be a safe and effective candidate in cadmium remediation process being efficient in metal sequestering, restoring cellular damage exerted by Cd(II) exposure, without altering cellular physiological activity.

## 1. Introduction

Metals represent one of the most widespread environmental contaminants. Among them, cadmium (Cd) is a persistent, non-essential metal coming from anthropogenic activities and natural processes and it is present in all the environmental matrices [1,2]. Cadmium enters the aquatic environment from diffuse and point sources, it is estimated that weathering and erosion contribute in 15,000 tons of cadmium every year while atmospheric fall-down (of anthropogenic and natural emissions) is estimated to contribute between 900–3600 tons to the global aquatic environment [3]. Cadmium concentration in industrial effluents may vary from 0 to 1000 mg L^−1^, whereas that in municipal waste waters is commonly lower than 0.01 mg L^−1^ [4]. Although drinking water usually contains very low concentrations of cadmium (ranging from 0.00001 mg L^−1^ to 0.001 mg L^−1^), levels up to 0.01 mg L^−1^ have been sometimes reported. In polluted areas, well-water cadmium concentration could exceed 0.025 mg L^−1^ [5,6]. On the other hand, speaking in terms of threshold values, while the European Union directive posed it 0.005 mg L^−1^ for drinking water [7], other countries like Brazil allowed the threshold to be set 0.01 mg L^−1^ for freshwater [8]. Moreover, besides smoking habit and alcohol consumption, diet plays an important role in humans being vegetables able to accumulate cadmium whose environmental levels need to be maintained under control [9].

Since 1993 the International Agency for Research on Cancer has classified cadmium and cadmium compounds as human carcinogens [10] and the contamination of surface and ground waters by cadmium represents a serious environmental problem which involves human health. An increased risk of cancer, including bladder cancer, was found to be statistically linked with cadmium contamination [11], thus indicating the development of remediation technology for the reduction of Cd content in freshwaters is highly recommended.

Cadmium carcinogenesis mechanisms seem to include oxidative DNA damage and subsequent apoptotic resistance, epigenetic DNA methylation status changes, aberrant gene expressions [12], and DNA repair systems interference [13]. Moreover, a possible dysfunction of mitotic apparatus resulting from incorrect segregation of the chromosomes at anaphase has been suggested [1].

In this context, the development of a sustainable methodology to safely remove cadmium from contaminated waters deserves much attention. Techniques of nano-remediation, which is the application of nanotechnology and the use of engineered nanomaterials (ENMs) to clean contaminated environmental matrices, including groundwater and wastewaters [14], can be considered the most appropriate tools.

For this purpose, the selected nanomaterial needs to be completely reliable, not exerting any degree of toxicity towards biota [15], thus overcoming the problems related for example with the use of zero valent iron (nZVI) [16,17] after injection into aquifers for in-situ remediation. The use of nZVI has shown great potential in the remediation of contaminated water [18], but it has also underlined its reactivity in the remediation of highly polluted areas, including toxic effects during environmental applications. Carbon nanotubes, nano-zinc and nano-titanium oxides have been tested in remediation processes [19,20,21], but conflicting results have been reported concerning their potential ecotoxicity [22]. For these reasons, sustainable nanomaterials, developed from renewable sources, arouse growing interest for their application in nano-remediation [22,23] being effective, biodegradable, and biocompatible. Among all the renewable sources, polysaccharides (and cellulose in particular) are emerging as starting materials for a wide plethora of applications, including the synthesis of engineered nanomaterials (ENMs) for environmental remediation [23,24,25].

In this context, a two-step protocol for the synthesis of a novel family of nanocellulose-based ENMs was set up. Among their features, these ENMs possessed superb performance in the adsorption of organic and inorganic pollutants. They were obtained by the cross-linking of 2,2,6,6-tetramethyl-piperidine-1-oxyl (TEMPO)-oxidized cellulose nanofibers (TOCNF) [26] with branched polyethyleneimine (bPEI), affording nanostructured cellulose nanosponges (CNS) [27].

These materials possess a 2D sheet-like morphology and are characterized by a high micro-porosity, as revealed by scanning electron microscopy (SEM) [28] and microcomputed tomography (µCT) analysis [27]. More interestingly, CNS also shows a nano-porosity, as revealed by in-depth small angle neutron scattering (SANS) and Fourier Transform Infrared Spectroscopy (FT-IR) studies [29], and can be also easily functionalized to confer the material additional properties, including ion sensing [30]. The high porosity allows to favor the contacting between metal ion analytes and active sites of the sponge, consisting in the cooperative chelating action of vicinal amino groups. To encourage the applicability of CNS as adsorbent materials for decontamination of heavy metal polluted waters, their environmental safety (ecosafety) was improved by exploiting an eco-design approach consisting into the combination of life cycle assessment (LCA) and environmental risks assessment [31,32,33]. In these works, the synthetic protocol was revised as function of the results achieved by a laboratory scale LCA study [31] and by their impact on marine biota (*Dunaliella tertiolecta* and *Mytilus galloprovincialis*), while employed for sea decontamination [31,32].

For the first time, in the present work we aimed to evaluate the safeness and efficacy of CNS when employed as adsorbent material in remediation of heavy metal contaminated freshwater by studying cellular responses in a freshwater organism.

The sedentary bivalve *Dreissena polymorpha* (zebra mussel) has been widely used to study the effects of classical and emerging contaminants due to its high filtration rate, suitable size for in vivo laboratory exposure, and maintenance [34].

The sensitivity of zebra mussel hemocytes to genotoxic compounds has been demonstrated through the induction of DNA strand breaks and DNA adducts, the increase of micronuclei and nuclear abnormalities [35,36] related with the level of contaminants in water [1,37].

In the present study, zebra mussel was selected as a biological model to evaluate the safety of CNS for freshwater organisms and to test CNS efficacy in the removal of Cd(II) from a laboratory freshwater environment, with consequent restoration of physiological cellular functions in exposed organisms.

DNA integrity loss, cellular proliferation, and chromosomal damage associated to CdCl_2_ exposure were investigated in *D. polymorpha* hemocytes to evaluate the remediation capacity of CNS and to ascertain their safeness at a cellular level.

## 2. Materials and Methods

### 2.1. Chemical Reagents

CdCl_2_ (CAS 7790-78-5) and Giemsa were purchased from Carlo Erba (Milano, Italy). Cotton linter cellulose was kindly provided by Bartoli Spa (Capannori, Lucca, Italy) paper mill. Cadmium analytical standard, low melting agarose, normal melting agarose, Neutral Red, PBS, and all the other reagents were purchased from Sigma Aldrich (Milano, Italy).

### 2.2. Preparation and Characterization of the Cellulose Nanosponges (CNS)

CNS were synthesized by reproducing a standardized protocol previously reported [28,31]. This procedure includes (a) the production of cellulose nanofibers, by means of TEMPO/NaClO/KBr mediated oxidation of cotton linters, (b) their cross-linking with 25 KDa branched polyethyleneimine (bPEI), (c) a freeze-drying process and a thermal treatment, in order to obtain a nanostructured xerogel. The obtained CNS undergo a washing step prior the use.

**Figure 1:**
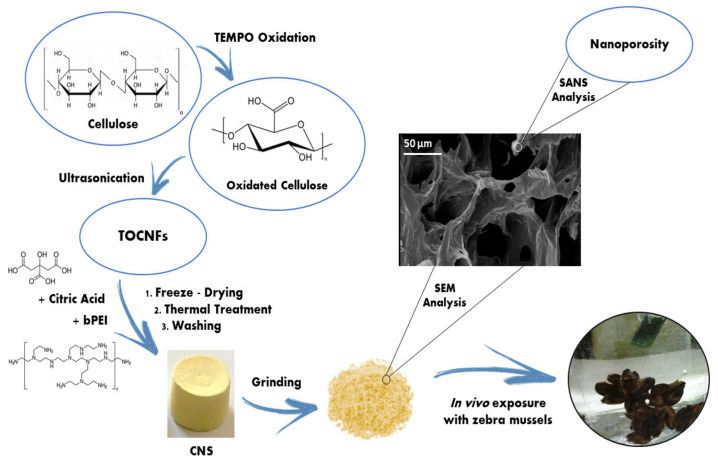
Schematic representation of synthetic and morphological aspects of cellulose-based nanosponge (CNS).

In detail, cotton linters cellulose (100 g) were minced and dispersed in deionized water (2 L) and then added to a solution (3.7 L) of TEMPO (2.15 g, 13.8 mmol) and KBr (15.42 g, 129 mmol) in demineralized water and kept under vigorous stirring. By means of two dropping funnels, NaClO (12.5% *w*/*w* aqueous solution, 437 mL) was added dropwise to the mixture, while sodium hydroxide solution (4 M) was added in order to maintain the pH value in the range of 10.5–11. The reaction was maintained under stirring for 16 h, and then acidified to pH 2 with concentrated HCl (37% *w*/*w* aqueous solution). TEMPO oxidized cellulose (84 g, 84% yield) was recovered as a white precipitate which was collected by filtration and washed extensively with deionized water (5 × 2 L) and acetone (2 × 0.5 L). The oxidation degree was evaluated by titration of the carboxylic groups introduced on the cellulose fibers using a NaOH solution and phenolphthalein as colorimetric indicator (1.5 mmol g^−1^ of -COOH units). After drying, TEMPO-oxidized cellulose was suspended in water, NaOH was added (1 equivalent respect to carboxylic acids), and the dispersion was ultrasonicated at 0 °C (Branson Sonifier 250, Branson Ultrasonic SA, Carouge, Switzerland), 6.5 mm probe tip, 20 kHz in continuous mode, output power 50%) until a clear solution was obtained, indicating the complete defibrillation of TEMPO-oxidized cellulose into nano-dimensioned fibers (TOCNF). An excess of HCl (aq, 1 M) was then added, the precipitate was collected by filtration and further washed on the filter with deionized water until the filtrate reached a pH 6–7. TOCNF were dispersed (2 % *w*/*v*) in an aqueous solution containing bPEI and citric acid (mass ratio 1:1:3.53 respectively), then the mixture was sonicated for 10 min before being transferred into a 24-well plate as mold, frozen at −80 °C and freeze-dried for 48 h, affording the corresponding xerogels which were removed from the mold and thermally treated in an oven (103 °C, 12–16 h). The resulting sponge-like materials (Figure 1) were ground in a mortar (particles sizes range 50–400 μm, maximum distribution 130 μm) and washed with water (6 times, 1 h contact time for each cycle).

### 2.3. Sampling and Maintenance Condition

Independent experiments were conducted in freshwater aquaria for the preliminary CdCl_2_ dose-exposure test and for experiments in which CdCl_2_ contaminated artificial freshwater was treated with CNS. Adult specimens of *D. polymorpha* (medium valves length 2 ± 0.5 cm) were collected from Bilancino Lake, a pristine area in Tuscany (Florence, Italy) and carried to the laboratory in lake original water. For the acclimatization period mussels were placed in a 10-L aerated tank with artificial freshwater (AFW) obtained by mixing distilled (50%) and dechlorinated tap water (50%) 5 days before preliminary dose-exposure experiment and 2 days before the second set of experiments. Water temperature was 18 ± 1 °C and a natural photoperiod was maintained, pH values were 8.04 ± 0.10 for preliminary dose-exposure experiments and 8.05 ± 0.15 for experiments with CNS.

### 2.4. In Vivo Exposure

After an acclimatization period, mussels were placed on glass sheets suspended in small glass aerated aquaria. For each treatment tank, at least 30 zebra mussels were exposed to the experimental time of 48 h. Zebra mussels were not fed during the experiments, and only specimens that were able to re-attach themselves by their byssus filaments on glass sheets immersed in water were used for the research as suggested by Binelli and collaborators [36]. Mussels were preliminarily exposed to 0.005; 0.01; 0.05; 0.1 mg L^−1^ CdCl_2_ for 48 h and to 7 days in order to set up sub-lethal concentration to be used for the CNS efficacy exposure study. The lowest dose was selected because established by United States Environmental Protection Agency (US EPA) as a maximum contaminant level for cadmium in drinking water [38] while the highest one is considered representative of highly impacted sites. CdCl_2_ was dissolved in distilled water and a stock solution was prepared. These preliminary studies indicated that a loss of DNA integrity in *D. polymorpha* hemolymph was observable after 48 h exposure, starting from 0.005 mg L^−1^ and from 0.01 mg L^−1^ after 7 days displaying cytotoxicity only at the highest dose of 0.1 mg L^−1^ (data not shown). For this reason, in order to work with environmentally realistic exposure levels, the CdCl_2_ dose selected for the experiments resulted to be 0.05 mg L^−1^, being genotoxic but not cytotoxic (cytotoxicity appeared at 0.1 mg L^−1^). Moreover, the dose of 0.05 mg L^−1^ (53.7 ± 0.5 µg L^−1^ nominal dose) allowed to assess CNS activity at a cadmium concentration which can be considered environmentally realistic in polluted sites [1], even avoiding mussel valves’ closure with consequent cessation of filtering activity, a general defense mechanism occurring in mussels after exposure to very high concentrations pollutants, higher than 0.1 mg L^−1^ for Cd(II) concentrations [39,40]. To assess CNS ecosafety, i.e., absence of toxicity in freshwaters, and CdCl_2_ adsorption efficacy, zebra mussels were in vivo exposed for 48 h to the following groups in AFW: CdCl_2_ 0.05 mg L^−1^ (Cd(II)), CdCl_2_ 0.05 mg L^−1^ after treatment with CNS (Cd-t CNS), AFW after treatment with CNS (CNS). Mussels in AFW were used as controls (AFW). The ratio of CNS able to adsorb Cd(II) from freshwaters was set up at 1.25 g CNS per L of AFW, based on our previous studies [31,33]. In particular, CNS samples were incubated with AFW for 2 h at room temperature, under vigorous magnetic stirring, reproducing the treatment process normally applied to contaminated water matrices. Mixtures were then filtered at 0.45 µm to remove CNS powder and the conditioned AFW was used for zebra mussels during the in vivo exposure study. The effects of CdCl_2_ and CNS-treated AFW, alone and in combination, were evaluated in zebra mussel hemocytes gently aspirated from the posterior adductor muscle sinus, processed for Comet and Cytome assays [41].

### 2.5. Cadmium Concentration in Water

Water media samples were taken from each tank after 48 h and stored at 4 °C, for chemical analyses. Cadmium concentrations in AFW were determined by means of Inductively Coupled Plasma-Optical Emission Spectrometry (ICP-OES) using a Perkin Elmer Optica 8300 (Perkin Elmer, Waltham, MA, USA), equipped with a CrossFlow nebulizer and a Scott Spray Chamber, followed by a standard quartz torch. The instrument calibration was performed by dilution of a cadmium analytical standard (Honeywell FLUKA^TM^, Fisher Scientific Italia, Rodano, MI, Italy), with MilliQ^®^ water obtaining 5, 10, and 50 μg L^−1^ solutions, to each analyzed samples Y (2 mg L^−1^) was added as internal standard.

### 2.6. Viability Assessment

The Neutral Red Retention Time (NRRT), used extensively as convenient and rapid measurement of cell viability [42], was performed to discriminate the healthy cells, whose lysosomes take up and retain longer the dye (neutral red), from damaged cells unable to keep the stain inside the organelles.

NRRT was set up according to Guidi and coworkers [43]. Briefly, 10 µL of hemolymph for each sample were incubated on a polylysine pre-coated microscope glass slide with neutral red working solution (0.1 mg mL^−1^). Granular hemocytes were examined to light microscope at 15 min intervals, for up to 180 min to evaluate the time at which 50% of cells had leaked to the cytoplasm the dye previously trapped by lysosomes. The Comet assay was performed only with cell population that showed a viability >90% to avoid false positive results.

### 2.7. Comet Assay

The Comet Assay was performed on hemocytes from 10 specimens per tank at the end of the exposure, according to Singh and co-authors [44] with slight modifications [43]. Briefly, cells were embedded in 0.5% low-melting agarose (LMA), spread onto microscope slides pre-coated with 1% normal-melting agarose (NMA) and covered with a further layer of LMA 0.5%. Slides were dipped into a lysing solution (NaCl 2.5 M, Na_2_EDTA 100 mM, Trizma Base 10 mM, 10% DMSO, 1% Triton X-100, pH 10) and kept overnight at 4 °C in the dark, in order to solubilize cell membranes and cytoplasm. Successively, slides were treated with alkali (NaOH 300 mM, Na_2_EDTA 1 mM, pH > 13) for 10 min and placed in a horizontal electrophoresis apparatus. Electrophoresis was performed for 5 min at 25 V and 300 mA. After the run, slides were neutralized with Tris-HCl (0.4 M, pH 7.5), stained with 100 μL ethidium bromide and observed under a fluorescence microscope (400×). The amount of DNA damage was quantified as the percentage of DNA migrated into the comet tail (tail DNA) [45], using an image analyzer (Kinetic imaging, Ltd., Komet, Version 5, Software for Live Cell Imaging, Tissue and Structure Quantification, Cytogenetics and Toxicology, Stereology. Bromborough, Wirral, Merseyside CH62 3NY, UK, 2005). At least 50 nuclei per slide and 2 slides per sample were scored, for a total of 100 nuclei per organisms and the mean calculated. At least 5 organisms per treatment were analyzed.

### 2.8. Cytome Assay

**Figure 2:**
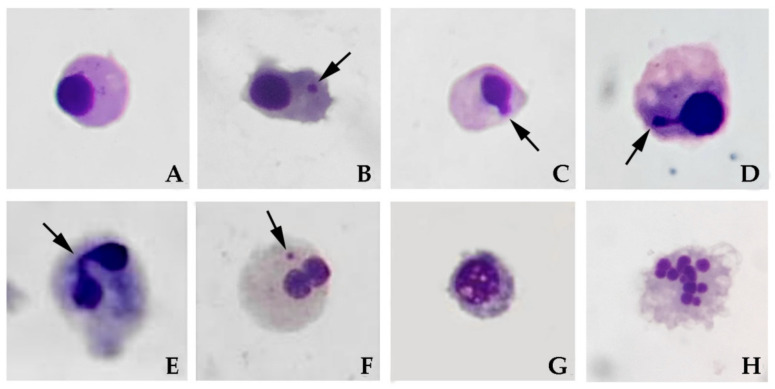
Hemocyte nuclear abnormalities in *D. polymorpha*. (**A**) Mononucleated healthy cell; (**B**) hemocyte displaying a micronuclei (MN); (**C**) hemocyte with a nuclear bleb (BL); (**D**) hemocyte with a nuclear bud (NBUD); (**E**) Binucleated (BN) hemocyte with nuclear bridges (NPB); (**F**) BN with a MN; (**G**) vacuolated cell; (**H**) apoptotic cell.

The genotoxic effects were evaluated at a chromosomal level by the micronuclei and nuclear abnormalities frequency assessment. The Cytome assay was carried out on hemocytes according to Guidi and co-authors [43]; 900 µL of PBS were added to100 µL of hemolymph, then centrifuged for 10 min at 1000 rpm. Cell pellet was prefixed for 20 min in a 5% acetic acid, 3% ethanol, 92% PBS, then centrifuged for 10 min at 1000 rpm. The supernatant was removed and 1 mL fixative (7:1, ethanol:acetic acid) was added to the suspended pellet; this process was repeated twice. After the last fixation cells were centrifuged, spread onto microscope slides (two slides per mussel), air dried and stained with 3% Giemsa solution for 10 min. Cells with well-preserved cytoplasm per specimen were scored (500 per slide) at light microscope to determine the frequency of micronuclei (MN) and nuclear abnormalities [46] according to the following criteria proposed by Fenech [47]. The presence of cells with morphologically altered nuclei (nuclear blebs (BL), nuclear buds (NBUD), notched nucleus (NT), lobed nucleus (LB)), and apoptotic cells (APO) were scored on the same slides in parallel and collectively reported. The frequency of total nucleus abnormalities (NA), was also evaluated in mussel hemocytes and binucleated cells (BN), with or without nuclear bridges (NPB), were scored for proliferation activity (Figure 2). Ten specimens, two slides per specimens, 500 cells per slide were scored for each experimental group.

### 2.9. Statistical Analysis

Results obtained are represented as mean ± SE from at least 5 specimens. Data were analyzed by the multifactor analysis of variance (MANOVA) or multiple regression analysis (MRA). The multiple range test (MRT) was performed in order to detect differences among experimental groups. For all data analysis, statistical significance was set at *p* < 0.05.

## 3. Results and Discussion

### 3.1. CNS Synthesis and Characterization

The aim of the present work was to assess the efficacy and the safety of specifically synthesized polysaccharide based nanosponges to prevent cadmium-induced genotoxicity in freshwaters in the framework of a bigger project on nanoremediation (“Nanomaterials for Remediation of Environmental Matrices associated to Dewatering”, POR CReO FESR 2014–2020). Concerning results about CNS synthesis and characterization, CNS were produced according to the protocol reported in Figure 1. In a first step, the oxidation of native cellulose by means of TEMPO/NaClO/KBr system leads to the partial conversion of C6 alcoholic groups present on the glucopyranose units to the corresponding carboxylic acids [26]. The final content of acidic groups resulted to be 1.5 mmol g^−1^, which was considered a limit value, as further oxidation would lead to partial depolymerization of cellulose chains. Carboxylic moieties, at basic pH, underwent deprotonation, promoting the electrostatic repulsion among single nanofibers (TOCNF), which could be filtered and recovered. The second step in the synthetic procedure consisted into mixing TOCNF and bPEI in deionized water in a 1:1 weight ratio. In order to increase the content of carboxylic groups and to further increase cross-linking, 18% of citric acid (CA) respect to primary amino groups of bPEI was added to the mixture. This formulation is the result of an eco-design performed since the early stage of production was at a laboratory scale [32]. The obtained hydrogel underwent lyophilization and then heating at about 100 °C, affording CNS, whose structural and chemical stability was derived by the formation of amide bonds between the carboxylic groups of TOCNF and the primary amines of bPEI, as evidenced in a previous work by FT-IR analysis (-C=O stretching of the amide bonds at 1664 cm^−1^) [28]. ^13^C CP-MAS solid-state NMR and elemental analysis also confirmed the role of CA in better fixing bPEI molecules [28], the latter playing a crucial role in the adsorption process by chelating the metal ions thanks to the multiple amino groups. CNS were grinded in a mortar before use, in order to increase sorption performance, by facilitating the diffusion of ions in the network [32]. Microcomputed tomography (μ-CT) analysis also revealed that CNS are characterized by a high porosity (70–75%), with pore sizes in the range of 10–100 μm, as evidenced by scanning electron microscopy (SEM) [28]. Pores are formed during the freeze-drying process, and are characterized by a 2D sheet-like morphology, often reported in literature for cellulose-based aerogels obtained following this procedure. This approach also prevents the formation of occlusions, guaranteeing a complete diffusivity of analytes in the network [48]. Besides the evidence of micro-porosity, a small angle neutron scattering (SANS) analysis of water nano-confinement geometries in CNS allowed to evidence the presence of nano-pores, measuring a short-range correlation length in a range between 25 and 35 Å [29]. More recently, the FTIR-ATR investigation of H_2_O and D_2_O in CNS revealed a supercooled behavior of these molecules, further supporting the concept a nano-confinement for water, and consequently the presence of nanopores in the network. Due to all these characteristics CNS represents a sustainable cellulose nanomaterial, developed from renewable sources, which appears to be eligible for nano-remediation strategies in heavy metal polluted environments.

As cadmium is a human carcinogen, its contamination of surface and ground waters represents a serious environmental problem, even if the mechanism of Cd-induced carcinogenesis is still under discussion. It was proposed that it is multi-factorial [49], and an important role may be played by the indirect increase of reactive oxygen species and the disruption of the cellular antioxidant system [12,50,51], leading to oxidative stress induction [52], which, in turn, causes lesions to the DNA, including potentially lethal DSBs [13]. It also competes with Zn (II) in cellular components and binds to the DNA bases, giving rise to single-stranded DNA breaks [53,54]. While the European Union directive posed 0.005 mg L^−1^ of cadmium as a threshold for drinking water, other countries allowed higher thresholds to be set for freshwater. For this reason, the ability of harmless polysaccharide based nanosponges to reduce both Cd(II) concentration in water and Cd(II)-induced genotoxicity in freshwater organisms was investigated.

### 3.2. Cadmium Concentration in Water

In order to assess the efficacy of cellulose-based nanosponges in removing Cd(II) from freshwater together with their safety evaluation for organisms, exposure experiments to cadmium contaminated AFW treated with CNS (Cd-t CNS) were carried out. ICP-OES analysis on exposure waters showed the strong Cd(II) adsorption capability of CNS in freshwater. Table 1 reports the results of ICP-OES analysis on all the waters to which zebra mussels have been exposed. In AFW and AFW treated with CNS (CNS) the Cd(II) concentration resulted negligible. On the contrary, artificially Cd(II) polluted AFW treated with 1.25 g L^−1^ CNS (Cd-t CNS) showed a remarkable decrease of Cd(II) concentration (up to 88.8%) when compared to artificially Cd polluted AFW (Cd(II)).

This result was in line with what was recently found in pure water [27] and in artificial sea water [32,33]. It is interesting to note how starting from 0.05 mg L^−1^, a dose associable with high contaminated rivers [55], the residual final cadmium concentration found in waters treated with CNS was 0.0060 ± 0.0001 mg L^−1^, the same order of magnitude posed by the European Union directive for drinking water (0.005 mg L^−1^).

### 3.3. Cellular Responses

Concerning biological responses water samples pretreated with CNS alone (CNS) or in combination with 0.05 mg L^−1^ CdCl_2_ (Cd-t CNS), they did not result as cytotoxic in terms of lysosome membrane stability, always displaying values of Neutral Red Retention Time above 180 min. After both the exposures to CNS alone and cadmium contaminated AFW treated with CNS, data were statistically comparable to control levels also in terms of DNA integrity.

Cadmium per se is weakly genotoxic [13], and one of the advantages of the Comet assay is its demonstrated sensitivity for detecting low levels of DNA damage and in its alkaline version it is capable of detecting DNA single-strand breaks (SSB), alkali-labile sites (ALS), DNA-DNA/DNA-protein cross-linking, and SSB associated with incomplete excision repair sites [56]. Moreover, a negative association has been often showed between oxidative stress and DNA primary damage detected by Comet assay [57].

**Figure 3:**
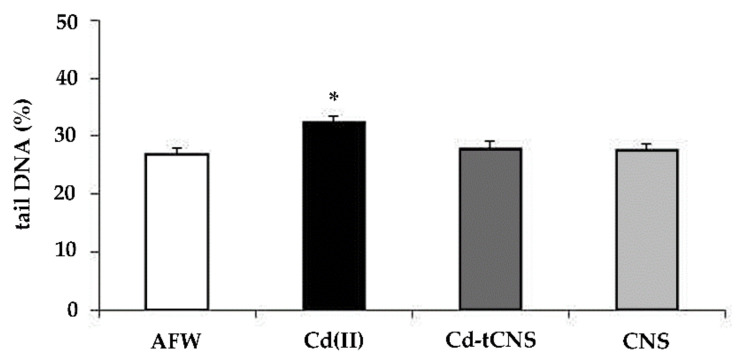
DNA primary damage (% tail DNA) in zebra mussel hemocytes after 48 h of exposure to the following experimental groups: AFW (control); Cd(II) (0.05 mg L^−1^ of CdCl_2_ in AFW); Cd-t CNS (CdCl_2_ 0.05 mg L^−1^ contaminated AFW treated with CNS); CNS (AFW treated with only CNS). Results are shown as mean ± SE. (*) indicates significant differences respect to the control group (AFW) (*p* < 0.05).

Zebra mussel hemocytes collected from the tank containing AFW exposed to polysaccharide-based nanosponges did not show any loss of DNA integrity. On the contrary, CdCl_2_ 0.05 mg L^−1^ induced a statistically significant increase (*p* < 0.05) of DNA primary damage in zebra mussel hemocytes compared to controls. In specimens exposed to Cd-t CNS the DNA damage induced by CdCl_2_ 0.05 mg L^−1^ turned down to control level (Figure 3). Interestingly, DNA integrity loss was found to be related to cadmium nominal concentration (c.c. 0.46; R^2^ = 21.7; *p* < 0.001).

Although our background level of DNA damage in control mussels did not range among the recommended values suggested by Tice and co-authors [56], the results obtained from the control tank were able to discriminate the exposure effects. In literature, high background levels obtained by the Comet assay were reported in *D. polymorpha* as well as in other aquatic organisms [58] and baseline levels of DNA damage in mussel hemocytes seem to be correlated with animal maintenance temperature; resulting 4 °C to be the optimal experimental condition for lower Comet assay basal results [59]. However, 4 °C was not selected in the present work because it was not representative of natural water lake temperature required by our experimental design which aimed, instead, to recreate environmental realistic conditions to test CNS performance and potential cellular toxicity. On the other hand, we cannot exclude that results obtained from AFW experimental group might have been modulated by other factors such as water quality of sample site and/or origin population genetic background. CNS thus resulted to be not genotoxic in terms of DNA primary damage, speaking in favor of it being a harmless material for biological systems. The significant loss of DNA integrity of zebra mussel hemocytes induced by Cd was completely restored in organisms exposed to Cd-t CNS paralleling the observed reduction of cadmium bioavailability in the water, thus indicating CNS efficacy in Cd removal.

**Figure 4:**
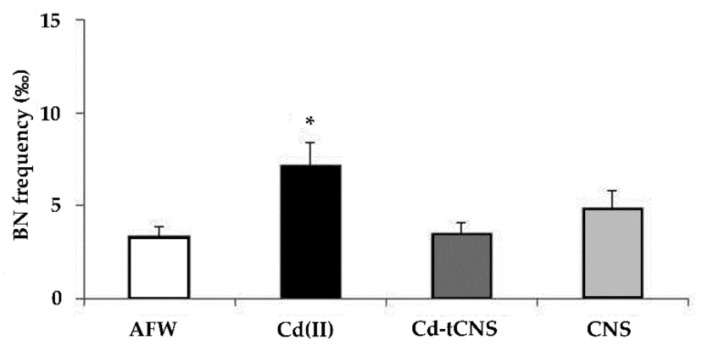
Frequency (‰) of binucleated cells (BN) in zebra mussel hemocytes after 48 h of exposure to the following experimental groups: AFW (control); Cd(II) (0.05 mg L^−1^ of CdCl_2_ in AFW); Cd-t CNS (CdCl_2_ 0.05 mg L^−1^ contaminated AFW treated with CNS); CNS (AFW treated with only CNS). Results are shown as mean ± SE. (*) indicates significant differences respect to the control group (AFW) (*p* < 0.05).

Besides the disruption in cellular signal transduction [60], cell proliferation activity appears to be affected by cadmium exposure [61] and our data seem to confirm in zebra mussel hemocytes what was reported for human and mammalian cell lines [62,63,64]. The dose selected for CdCl_2_ treatment (0.05 ppm) was able to induce proliferation activity evaluated in terms of binucleated cell frequency increase (*p* < 0.05) after 48 h exposure. Interestingly, after 2 day-exposure the frequency of binucleated hemocytes turned down to control level in specimens exposed to cadmium in AFW treated with CNS (Cd-t CNS) (Figure 4).

Cadmium was actually found to stimulate DNA synthesis and cell proliferation at low concentrations in mammalian cell lines [54], leading to relevant implications for carcinogenesis processes. An increased level of binucleated gill cells has been also observed in zebra mussel after 5 days of exposure and proposed to be related to an inhibition of cytokinesis activity more than a Cd proliferative effect [1]. Alteration of cytoskeleton function, resulting in reduced phagocytic processes, has been observed in *D. polymorpha* hemocytes [2] after 5 × 10^−4^ M Cd exposure. Moreover, an alteration in actyne fibers, resulting in altered hemocyte morphology, with reduced pseudopods formation, was reported after cadmium exposure in marine mussels [2,65,66]. In our study, the significant increase of binucleated cell frequency induced by Cd treatment was reduced to control levels in organisms exposed to cadmium contaminated AFW treated with CNS, showing that the Cd removal by CNS restored a physiological proliferation and/or cytokinesis activity. Among several mechanisms proposed to explain actin cytoskeleton alterations, an increase of ROS production plays an important role, which, in turn, might associate the two phenomena observed, i.e., loss of DNA integrity and cell proliferation increase.

**Figure 5:**
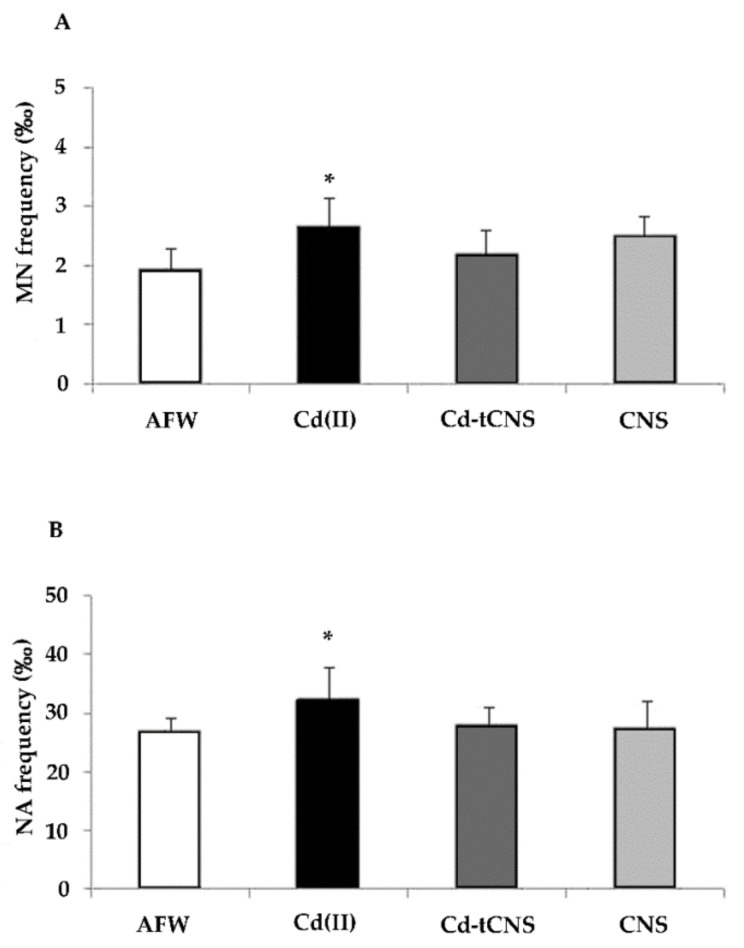
(**A**) Frequencies (‰) of micronuclei (MN) and (**B**) total nuclear abnormalities (NA) in zebra mussel hemocytes after 48 h of exposure to the following experimental groups: AFW (control); Cd(II) (0.05 mg L^−1^ of CdCl_2_ in AFW); Cd-t CNS (CdCl_2_ 0.05 mg L^−1^ contaminated AFW treated with CNS); CNS (AFW treated with only CNS). Results are shown as mean ± SE. (*) indicates significant differences respect to the control group (AFW) (*p* < 0.05).

Cd-induced biochemical changes may play roles in all the stages of carcinogenicity and the induction of oxidative stress in combination with decreased DNA repair can lead to DNA damage [54]. It has been proposed that Cd interferes with major DNA repair systems [13], leading to their inhibition [67,68], and unrepaired DNA lesions may give rise to chromosomal damage. The spontaneous MN frequency (mean ± SD) found in our preliminary studies (1.2 ± 1.1 for 2 days and 2.1 ± 1.05 for 7 days exposure, data not shown) is similar to the basal levels reported in zebra mussel hemocytes by Binelli and coauthors [36] (from 0 to 4). In the present study, Cd(II) exposure induced an increase of hemocyte MN basal level of 83%. AFW exposed to CNS did not cause any chromosomal damage in zebra mussel hemocytes, while CdCl_2_ 0.05 mg L^−1^ confirmed the statistically significant induction of cells displaying both micronuclei and total nuclear morphology abnormalities (Figure 5A,B). No induction of apoptotic cells was observed at all the experimental points.

**Figure 6:**
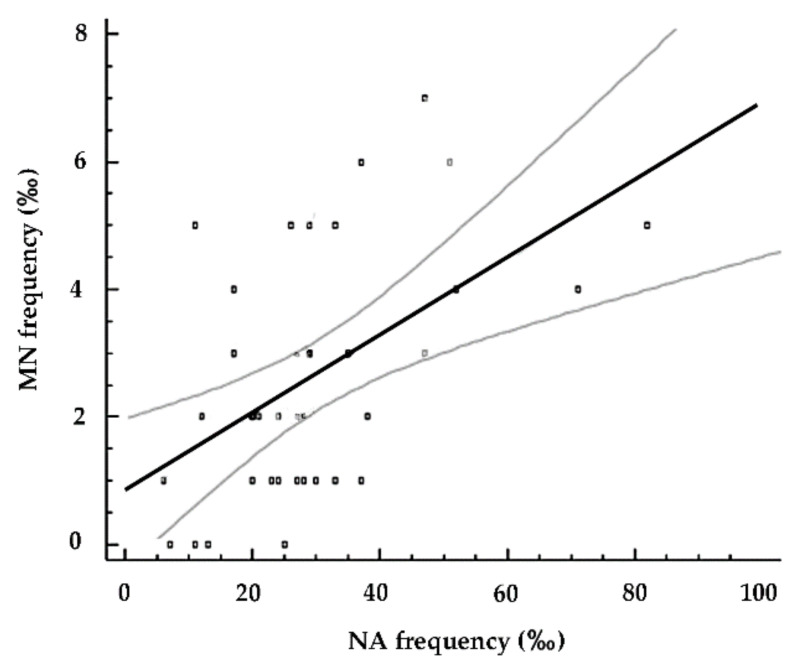
Relationship between MN and NA frequencies in zebra mussels from the different experimental conditions (control, Cd(II), Cd-t CNS, CNS). *r* = 0.52; R^2^ = 27.17; *p* < 0.001. Black line is the regression line, which represents the regression equation. Gray lines represent 95% of the confidence interval.

Mussels exposed to CNS alone and Cd-t CNS showed that Cd-induced MN and nuclear abnormality frequency increases, biomarkers associated with numerical and structural chromosomal mutations, were reduced by Cd removal activity of CNS, speaking in favor of the effectiveness of this newly synthetized nanosponge in restoring not only a Cd(II) induced repairable DNA damage, assessed by Comet assay, but also in reducing a consolidated chromosomal damage in freshwater organisms. Taking into consideration all the chromosomal damage, it is represented by both micronuclei, potentially coming from a possible dysfunction of mitotic apparatus, and nuclear abnormalities. NA include structural aberrations mainly representing the results of clastogenic events often induced by oxidative processes, like dicentric chromosomes (NPB), DNA repair system failure and gene amplification. In the present study, Multiple Regression Analysis also showed a moderate but significant positive correlation between the frequencies of micronuclei and total nuclear abnormalities (*r* = 0.52; R^2^ = 27.17; *p* < 0.001) (Figure 6), indicating the involvement of the whole chromosomal damage in response to cadmium-induced genotoxicity.

Our analyses also supported the use of hemolymph as a useful tissue to perform Cytome assay, allowing the analysis of samples from single individuals and not from pooled cells taken from different specimens as frequently applied [69].

The principal novelty of the present work relies on the efficacy of specifically designed eco-safe CNS for nanoremediation, which were found to efficiently remove cadmium from contaminated waters without inducing cellular alterations in a model freshwater organism. Data from literature concerning nano-remediation procedures mainly report cadmium removal from solid environmental matrices, like river sediments, by the use of nZVI nanomaterials [11,70], making less fitting any comparison with the present study. In such cases, the remaining concentration of Cd(II) in the aqueous solution was enough to induce strong deleterious effects on the mechanisms of cellular respiration [70]. Nano TiO_2_ sprays have been even used to reduce cadmium content in cowpea plant leaves [21] in order to reduce cadmium dietary intake, but the need of cadmium removal from freshwaters is still high and future research directions call for the assessment of CNS efficacy by using lower concentrations of cadmium, aiming to break down Cd(II) concentration close to zero level and to promote their potential large scale applications in different freshwaters and other environmental matrices.

## 4. Conclusions

We have proposed a cellulose-based nanostructured material derived from renewable sources as a valuable sorbent material for cadmium ions removal from freshwater. The high affinity for metal ions is ascribed to the chelating action of the amino-groups present in the inner structure, and derived from the bPEI polymer used for TOCNF cross-linking. The eco-designed material is characterized by a high micro- and nano-porosity, facilitating the contacting between the metal ions and the active sites of the sorbent. In our experimental model, this cellulose based nanosponge resulted to be a suitable safe candidate in cadmium remediation process being efficient in metal sequestering, without altering cellular physiological activity in freshwater organisms. Our data demonstrate, for the first time, the efficacy of CNS to carry out their remediation action in freshwater environment in terms of reduction of DNA primary and chromosomal damage induced by Cd to freshwater zebra mussel hemocytes. The efficacy of CNS was confirmed at a chromosomal level by the Cytome assay, which included the evaluation of different nuclear abnormalities, supporting the application of piscine Cytome assay [71] as a valuable tool for nano-remediation validating studies applied to freshwater models.

## Figures and Tables

**Table 1:** Cd(II) concentration (mg L^−1^) measured by plasma spectroscopy in treatment waters after 48 h of exposure to the following experimental groups: artificial freshwater (AFW) (control); Cd(II) (0.05 mg L^−1^ of CdCl_2_ in AFW); Cd-t CNS (CdCl_2_ 0.05 mg L^−1^ contaminated AFW treated with CNS); CNS (AFW treated with only CNS).

Experimental Group	Cd(II) mg L^−1^
AFW	<0.001
CNS	<0.001
Cd(II)	0.0537 ± 0.005
Cd-t CNS	0.0060 ± 0.0001
